# Presence of 15p Marker D15Z1 on the Short Arm of Acrocentric Chromosomes is Associated with Aneuploid Offspring in Mexican Couples

**DOI:** 10.3390/ijms20215251

**Published:** 2019-10-23

**Authors:** Sandra Ramos, Rebeca Rodríguez, Oscar Castro, Patricia Grether, Bertha Molina, Sara Frias

**Affiliations:** 1 Laboratorio de Citogenética, Instituto Nacional de Pediatría, 04530 Mexico City, Mexico; sera_ramos@yahoo.com.mx (S.R.); r-eb_ec-a@hotmail.com (R.R.); castrum_1981@yahoo.com.mx (O.C.); bertha_molina@yahoo.com.mx (B.M.); 2 Laboratorio DIAGEN, 05300 Mexico City, Mexico; pgrether@gmail.com; 3 Departamento de Medicina Genómica y Toxicología Ambiental, Instituto de Investigaciones Biomédicas, Universidad Nacional Autónoma de Mexico, 04510 Mexico City, Mexico

**Keywords:** chromosome polymorphisms, acrocentric chromosomes, satellite III, chromosome 15p, D15Z1, aneuploidy

## Abstract

Variation in the location of the 15p region D15Z1 is recognized as a polymorphism in several human populations. We used high-stringency Fluorescence In Situ Hybridization (FISH) to detect D15Z1 in a Mexican cohort. Here, we report the presence of extra D15Z1 sequences on the p-arm of acrocentric chromosomes other than 15 in two groups of Mexican couples, one with healthy offspring (*n* = 75) and the other with aneuploid offspring (*n* = 87), mainly trisomy 21. The additional D15Z1 polymorphism was significantly increased in individuals with aneuploid offspring (26.4%), in comparison to individuals with healthy offspring (14%). The most frequent acceptor chromosome of D15Z1 was chromosome 13p, followed by 14p, and finally, 21p. Our results show an overall frequency of 21.6% of this polymorphism in the Mexican population and suggest that its presence might be associated with the mis-segregation of other acrocentric chromosomes and aneuploid offspring. The high frequency of the polymorphism of the D15Z1 sequence on acrocentric chromosomes other than 15 suggests a sequence homogenization of the acrocentric p arms, related to the important function of the centromere and the nucleolar organization region, which flank satellite III DNA.

## 1. Introduction

The human acrocentric chromosomes are classified in group D, which includes pairs 13, 14, and 15, and in group G, which includes pairs 21 and 22. The study of acrocentric chromosomes has gained relevance given their involvement in clinically important cytogenetic alterations, such as: (a) aneuploidy, the leading cause of spontaneous abortions and congenital birth defects; (b) marker chromosomes, which are chromosomes that are not identified by banding pattern; and, (c) Robertsonian translocations, formed through the fusion of two acrocentric chromosomes with breakpoints mainly in the proximal short arms [1,2]. All three of these cytogenetic alterations are important for human health. Aneuploidy is present in approximately 10 to 30% of human zygotes, 50% of spontaneous abortions, and 0.3% of human newborns [3,4,5,6]. Importantly, acrocentric chromosomes have the highest rate of aneuploid events [7,8]. Close to 70% of marker chromosomes found in humans are derived from acrocentric chromosomes, most of them from chromosome 15 [9,10,11,12]. Finally, Robertsonian translocations are the most frequent chromosomal rearrangement in humans with a frequency of 1/1000 individuals [13,14].

Distributed across the short arms of the five acrocentric chromosomes are the ribosomal ribonucleic acid (rRNA) genes, in a region called the nucleolar organization region (NOR). The NOR is composed by repeated units of a transcribed region with a gene encoding 18S, 28S, and 5.8S rRNAs, and a non-transcribed spacer region. High amounts of rRNA are required for the biogenesis of ribosomes, which are the sites of protein synthesis; therefore, this region of our genome has the highest transcriptional rate and rRNAs are essential for life [15].

Despite the importance of the short arms of acrocentric chromosomes, very little information exists regarding their sequence. According to the International System for Human Cytogenomic Nomenclature (ISCN), acrocentric p arms are divided into three regions that are distinguishable under a microscope, short arms p11, stalks p12, and satellite p13 [16], all of them made up of highly repetitive elements. The p13 region is the most distal and contains repetitive TTAGGG telomeric sequences and proximally beta-satellite DNA, which consists of a basic monomer of approximately 68 bp that repeats in tandem and diversifies, forming higher order structures that generate subsets, one of which is shared among the p13 region of all acrocentric chromosomes [17]. The p12 region are the stalks, containing the NOR, which are composed of tandem repeats of the ribosomal DNA (rDNA) unit containing a 45 bp pre-rRNA coding sequence and spacer DNA carrying regulatory elements for rRNA transcription; each of the five acrocentric chromosomes has one NOR that can vary from 70 kb, equivalent to one rDNA repeat, to 6 Mb, equivalent to more than 140 copies of the rDNA unit [18]. The most proximal region p11 is composed by several types of highly repetitive DNA, such as satellites I, II, III, IV, and a beta-satellite region proximal to NOR. Satellite I is a 17–25 bp repeat unit, while satellites II and III are made of a variety of 5 bp repeat units forming clusters that are shared by more than one acrocentric chromosome [19]. 

**Figure 1:**
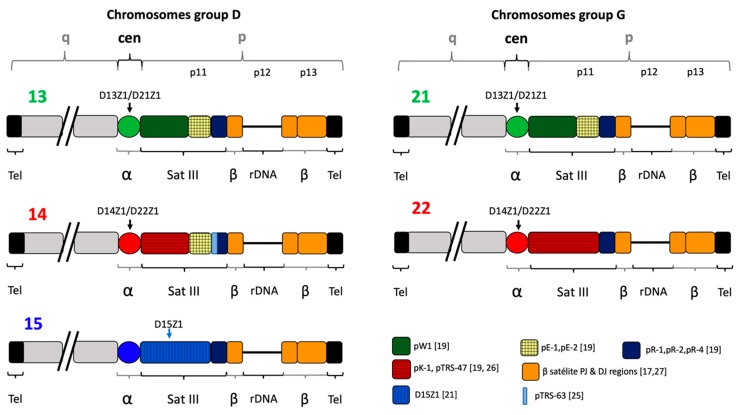
Schematic representation of the sequences on the short arms of human acrocentric chromosomes. Organization of β satellite DNA according to Floustakou et al. [25] and Waye et al. [17]. The region comprising Satellite III pR-1, pR-2, pR-4, perinucleolar β satellite PJ and DJ, NOR and telomeric regions are shared by all acrocentric chromosomes. The most variable region is that of the proximal satellite III, although chromosomes 13, 14, and 21, on one hand, and chromosomes 14 and 22, on the other hand, share sequences. The only chromosome-specific sequence is the satellite III subfamily D15Z1, typical of chromosome 15p. Organization of the subfamilies of satellite III DNA according to Bandyopadhyay et al. [19], Jarmuz et al. [26], Higgins et al. [21], and Choo et al. [27]. Arrows indicate regions that were analyzed by Fluorescence In Situ Hybridization (FISH) in this study.

The satellite III family is the most abundant family in the acrocentric p11 region and it is involved in the break points of the majority of Robertsonian translocations. Specifically, the proximally located subfamily pTRS47 has been directly implicated in the origin of the most common Robertsonian translocations, 13q14q and 14q21q, presumably due to the presence of common sequences that elicit recombination events resulting in a dicentric translocation [20]. Only one cluster of Satellite III repeats has been found to be chromosome-specific for 15p11, the D15Z1, which is composed by a consensus unit 5′AATGG3′ tandemly arranged. D15Z1 is a 1.8 kb fragment that is obtained by digestion with KpnI restriction enzyme and it is repeated approximately 3000 times per haploid genome [21] (Figure 1). It has been postulated that the D15Z1 region presents a high frequency of recombination or breaking events, since it is the principal site of the breakpoints forming dicentrics in Robertsonian translocations involving chromosome 15 [20]. In addition, the probe for the D15Z1 sequence frequently hybridizes on the p arms of acrocentric chromosomes other than 15, which generates an extra signal in the normal population [22,23,24]. This polymorphism has been postulated to be involved in chromosomal translocations [2], but little is known regarding its presence in the general population of diverse ethnic groups and about its possible role in the generation of chromosomal alterations. In this study, we evaluated Mexican couples with healthy offspring and couples who have had aneuploid offspring involving acrocentric chromosomes for the presence of D15Z1 on acrocentric chromosomes other than 15.

## 2. Results

### 2.1. Population Cohorts.

A total of 75 couples (150 individuals) with healthy offspring and 87 couples (174 individuals) with aneuploid offspring were studied, the latter included having children and/or miscarriages with aneuploidy of the acrocentric chromosomes; 83 couples had offspring with trisomy 21, three with trisomy 22 and one couple had offspring with trisomy 15. The average age in couples with aneuploid offspring was 32.2 ± 8.08 for fathers and 30.4 ± 7.3 years for mothers; the age of fathers and mothers with healthy children were 27.9 ± 5.8 and 25.9 ± 5.3 years, respectively. In addition, we studied 10 karyotypically normal descendants from couples with healthy offspring and 73 children with trisomy 21 born from couples with aneuploid offspring.

The GTG banding karyotype in peripheral blood revealed two abnormal karyotypes in each group of parents. In parents with healthy offspring, a mother showed a 4.3% monosomy for X chromosome and another mother was the carrier of a paracentric inversion of chromosome 8 in 100% of her cells. In the parents with aneuploid offspring, two fathers presented mosaicism, one with 4.6% of cells with trisomy 21 and the second with 6.9% of cells with monosomy X, who also presented an extra signal D15Z1 on 13p; both fathered children with non-mosaic trisomy 21 (Table 1). We considered the presence of these abnormalities to be non-related to the presence of aneuploidy in the offspring or associated to the presence of the studied polymorphism.

### 2.2. Frequency of Extra D15Z1 Region in the Different Groups of Study

**Figure 2:**
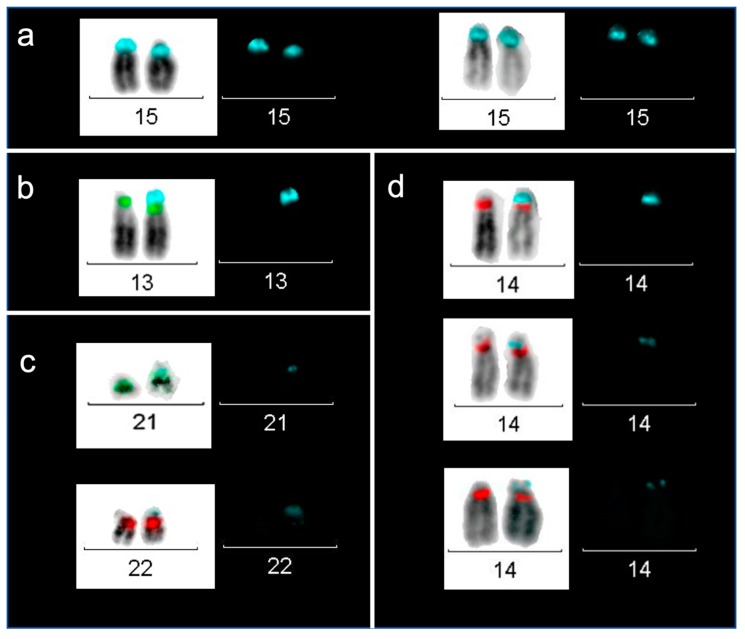
Hybridization of D15Z1 on acrocentric p arms of acrocentric chromosomes. We used probe D13Z1/D21Z1 in spectrum green, probe D14Z1/D22Z1 in spectrum red and probe D15Z1 in spectrum aqua. Left, merged images of 4’,6-diamidino-2-phenylindole (DAPI) bands in greys and triple-band filter fluorescence; right D15Z1 signal observed with aqua single-band filter. (**a**). Chromosomes 15 showing the normal D15Z1 signal; (**b**). One D15Z1 signal on chromosome 13p; (**c**). One D15Z1 signal on G chromosomes 21p and 22p; and, (**d**). One D15Z1 signal on chromosome 14p, and examples of the different sizes of the extra signal.

The D15Z1 region was searched by using a combination of FISH probes for D15Z1, D13Z1/D21Z1 and D14Z1/D22Z1, plus DAPI-banding, to identify the chromosome and the arm, where the additional D15Z1 was located on acrocentric chromosomes. D15Z1 signal was found in 100% of the studied individuals on both 15p11 regions. Overall, we found additional D15Z1 signals on other acrocentric chromosomes in 21.6% of individuals; approximately 75% of the additional signals showed a smaller size when compared with the normal signals that were observed on pair 15 (Figure 2). In all cases with an extra D15Z1 signal, it hybridized between the centromere and the NOR region of the acrocentric chromosomes. This polymorphism was never mosaic, since the totality of the analyzed cells within each case presented the same hybridization pattern. No D15Z1 signal was observed on any other autosome or sex chromosome in any of the analyzed samples.

The percentage of couples with an extra D15Z1 signal among the parents with clinically healthy children was 25.3%, whereas it increased to 44.8% in couples with aneuploid offspring (*p* = 0.06 Fisher’s Exact Test, two Tails). No difference in the incidence of maternal vs paternal carriers was observed and no significant difference was found when a correlation with the presence of the extra D15Z1 signal and advanced maternal age (>35 years old) was sought (Table 2). Both couple members presented an extra D15Z1 signal in three couples with healthy offspring and eight with aneuploid offspring.

Analysis per individual showed that, in total, 88/407 (21.6%) individuals presented the polymorphism. In the group of individuals with aneuploid offspring, the proportion of carriers was significantly higher, the presence of the additional D15Z1 polymorphism in individuals with aneuploid offspring was 26.4%, in comparison to individuals with healthy offspring 14% (*p* = 0.022 Fisher’s Exact Test, two tails) as compared with those with healthy offspring; among the individuals with the polymorphism, we found four carrier individuals with more than one extra D15Z1 signal in each group. Overall, we found the D15Z1 polymorphism in 20.7% across the 324 studied parents (Table 3) and 21.6% when we included the 83 studied children (Table 4).

The inheritance of the polymorphism was studied in the descendants from 10 out of 21 couples with healthy children and from 33 out of 39 couples with aneuploid offspring that accepted that their children participate in the study; 5/10 and 15/33 children inherited the polymorphism, which corresponds to the expected 50% of a normal segregation. In the group of aneuploid offspring, we identified 16 children with an additional D15Z1 signal distributed, as follows: eleven on 13p, three on 14p, two on 21p; three of them had two extra signals (Table 4). One child with 13pvar(D15Z1+) born from a couple without this polymorphism is the only apparent *de novo* case (we did not search for illegitimate paternity).

### 2.3. Localization of D15Z1 on Acrocentric Chromosomes other than 15.

**Figure 3:**
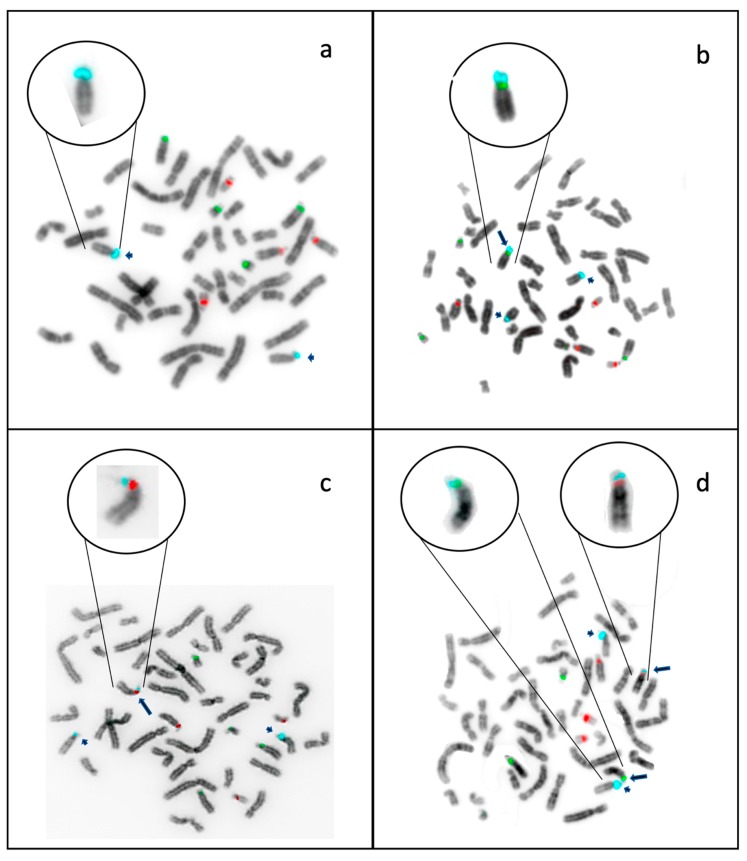
Metaphase plates with merged images of DAPI bands in greys and triple-band filter fluorescence. Fluorescent probes for D13Z1/D21Z1 in spectrum green, D14Z1/D22Z1 in spectrum red and D15Z1 in spectrum aqua; (**a**). metaphase from an individual without polymorphism, showing two D15Z1 signals; (**b**). metaphase from a carrier of 13pvar(D15Z1+), showing three D15Z1 signals; (**c**). metaphase from a carrier of 14pvar(D15Z1+), showing three D15Z1 signals; (**d**). metaphase from a carrier of two polymorphic chromosomes 13pvar(D15Z1+) and 14pvar(D15Z1+), showing four D15Z1 signals. Short arrows show the normal localization of the D15Z1 signal, long arrows show the polymorphic extra-signal.

In our study, an extra D15Z1 signal was always observed on the short arm of another acrocentric chromosome. We detected the presence of additional D15Z1 regions mainly on the acrocentric chromosomes of group D, including 13p and 14p (Figure 3). D15Z1 was less frequently located on acrocentric chromosomes of group G: eight individuals presented extra D15Z1 signals on 21p and only one in 22p (Table 4). 

**Figure 4:**
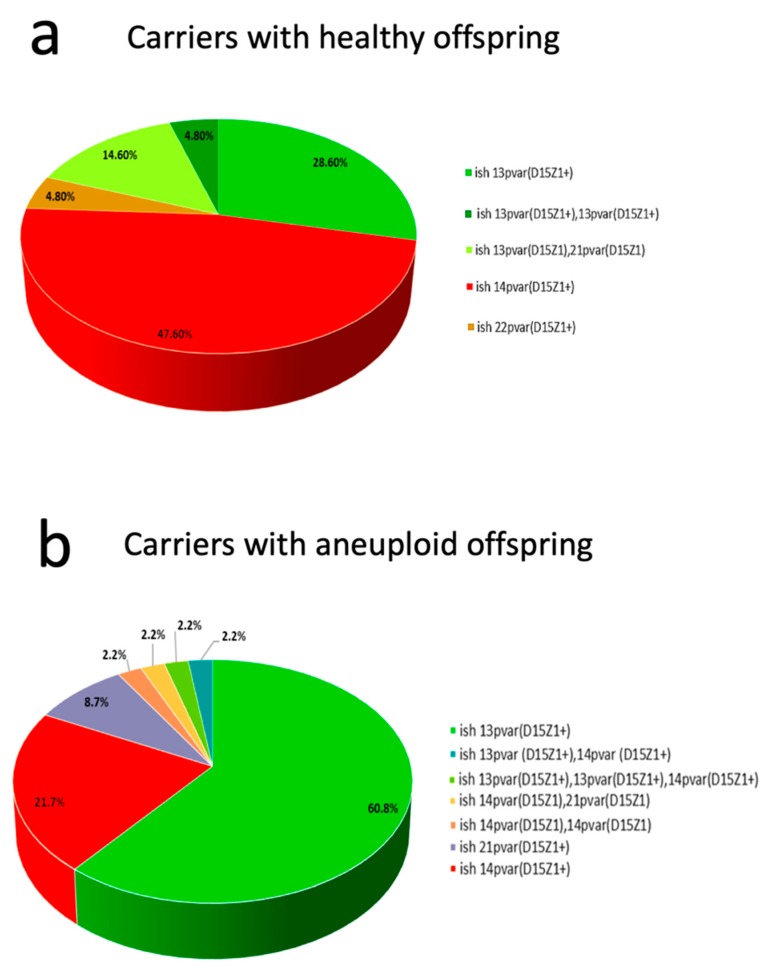
Overview of the frequency and location of the extra D15Z1 signals in carriers of the polymorphism. (**a**) In the carriers within the group with healthy offspring, chromosomes 13p, and 14p are the main acceptors of the D15Z1 chromosome region in similar proportion. Note that the proportion of 13p as an acceptor chromosome includes heterozygous, homozygous and compound heterozygous 13pvar(D15Z1+), 21pvar(D15Z1+) for a total of 48%. (**b**) In the group of individuals with aneuploid offspring, chromosome 13p is the main acceptor of D15Z1 with up to 65.2% of all the cases, which includes both heterozygous and homozygous individuals for 13pvar(D15Z1+), the 14pvar(D15Z1+) reaches 26% taking in account all variants.

The main D15Z1 acceptor chromosome varied, depending on the studied group. In couples with healthy offspring, the acceptor chromosomes were 13p and 14p in a very similar frequency; however, in couples with aneuploid offspring, >65% had the extra D15Z1 on 13p and only 26% on 14p. In twelve individuals, more than one extra signal was observed, but always on another acrocentric chromosome (Figure 4).

In our cohort, the frequency of heterozygous carriers for the 13pvar is 11.8%, and the frequency of homozygous carriers is 1.2%, while for the 14pvar, the frequency of heterozygous carriers is 7.9% and the frequency of homozygous carriers 0.25%. If we consider the 13pvar(D15Z1+) and 14pvar(D15Z1+) polymorphic variants as alleles, we found that 13pvar(D15Z1+) is outside the Hardy–Weinberg equilibrium in the parents of healthy children, mainly by an overrepresentation of homozygous individuals (Fisher’s Exact Test, two tails *p* = 0.048). On the other hand, when comparing the genotypic frequencies between the parents of healthy offspring versus the parents of aneuploid offspring, we found that the 13pvar(D15Z1) is associated with a higher risk of having aneuploid offspring (Armitage´s Trend Test *p* = 0.029).

## 3. Discussion

The investigation of the D15Z1 polymorphism began in 1991 with the study of Smeets et al., in a group of patients from a hospital in the Netherlands [22]. Using DA/DAPI (Distamycin A/4,6-diamino-2-phenyl-indole) staining, they detected the presence of the additional D15Z1 region on chromosomes 14p (6/127 individuals) and 13p (1/127 individuals) [22]. Shortly after, in a cohort of patients from the United Kingdom, Stergianou et al., using the satellite III probe D15Z1, found a frequency of 12/100 individuals carrying the polymorphism and, in all cases, the extra signal was on chromosome 14p [23]. In these two reports, the population studied consisted of patients with suspected chromosomal abnormalities. The largest cohort studied so far was reported by Cockwell et al., who analyzed the karyotypes of 1657 British individuals, 1336 of whom had a normal karyotype and 321 had altered karyotypes involving acrocentric chromosomes while using FISH with a D15Z1 probe at high stringency conditions. Their findings showed that 17.6% of individuals in the cohort had additional 15p signals on other acrocentric chromosomes, mostly on chromosome 14p (60%), followed by chromosome 13p (23%), with the remainder having the extra signal on chromosomes 21p, 22p, or multiple signals. Of note, they did not find differences among the studied groups, neither with normal or abnormal karyotypes [24]. In this work, we report, in the Mexican mestizo population, a frequency for the extra D15Z1 polymorphism of 21.6%, very similar to the frequency reported by Cockwell et al. However, at variance with the study of Cockwell et al., chromosome 13p was the main acceptor chromosome in our cohort. These results indicate that, in different ethnicities, such as the Caucasians or the Mexican mestizo population, the presence of an additional D15Z1 region on the short arms of non-15 acrocentric chromosomes exists as a polymorphism at a similar frequency.

In our study, we included two groups of Mexican couples, one with healthy offspring and the other with aneuploid offspring, mainly trisomy 21. We found two individuals with healthy offspring, with abnormal karyotype, without the D15Z1 polymorphism and with normal descendants. In addition, we found two interesting cases of abnormal karyotype among the individuals with aneuploid offspring. One of them is a father with 4.6% of cells with trisomy 21, and it is possible that mosaicism is also present in his germ cells, which would directly explain the aneuploid offspring. It has been observed that 5% of phenotypically normal parents of a trisomic 21 child are mosaics for trisomy 21 [28]. The other case is a father with 6.9% of cells 45,X, missing the chromosome Y, and it is a carrier of an additional signal D15Z1 on chromosome 13p. In this case, two possible factors may explain the aneuploidy in the father and in the child: (a) monosomy of X chromosome in mosaic has been described in couples with infertility and abortions, both being related to the presence of aneuploid embryos, suggesting an abnormal genetic control of chromosomal disjunction [29,30,31]; (b) the presence of the polymorphism in the father could increase the possibility of a translocation between chromosome 15p or 13pvar(D15Z1+) and the Yq chromosome. Several cases of translocation of D15Z1-pter to the heterochromatic region of the Yq chromosome have been reported [32,33,34,35]. It is possible that the known homology between a subdomain of DYZ1 and chromosome 15p [36,37] could favor the proximity of these chromosomes, increasing the possibility of a translocation [38] that generates an unstable chromosome that is prone to loss. These hypotheses deserve to be further investigated.

In our cohort, the presence of the additional D15Z1 polymorphism was significantly increased in individuals with aneuploid offspring (26.4%), in comparison to individuals with healthy offspring (14%). No correlation was found between the presence of the additional D15Z1 polymorphism and the parental gender or advanced maternal age (>35 years old), which is a well-known risk factor for aneuploid offspring [4]. In addition to advanced maternal age, meiotic recombination defects have been identified as factors predisposing to the non-disjunction of chromosomes. For example, a high rate of recombination events near the centromeres has been associated with the miss-segregation of chromosome 21 in young mothers [39]. A variant in the structure of the repeated sequences of the acrocentric short arms could affect chromosome pairing and recombination.

Although we found a significant association between the presence of additional D15Z1 signals and aneuploid offspring, before claiming that a true association between the presence of the polymorphism and an increased risk for chromosome trisomy exists, the following caveats need to be considered:

(a) in this study, only a minority of the additional D15Z1 signals was found on chromosome 21p, the chromosome that is involved in aneuploidy of the majority of the offspring.
(b) we detected similar frequencies for the D15Z1 polymorphism between both male and female progenitors, which rules out an exclusive association between the presence of this polymorphism and non-disjunction events during oogenesis. Non-disjunction events during oogenesis are widely known to be responsible for >90% of events leading to trisomy 21, as well as for other acrocentric chromosome trisomies [4].
(c) the frequency of additional D15Z1 signals in the aneuploid offspring group was 21.9%, which is similar to a previously reported cohort of aneuploid abortions that presented the variant in 17.7% of the products, without a significant difference with the healthy population [40], although in aneuploid descendants with the polymorphism, either born alive or aborted, the acceptor chromosome was mostly 13pvar(D15Z1).

The above considerations suggest that the presence of the D15Z1 variant might not be directly related to non-disjunction in the carrier couples. However, multiple risk factors for conceiving aneuploid offspring have been found, such as advanced age of the mother and grandmother, recombination defects, and exposure of the parents to chemicals, tobacco, or alcohol use [4,5,41]. Advanced maternal age is the best recognized risk factor, although more than 60% of aneuploid patients are born to young mothers [42]. In our study, the couples are mostly <35 years of age, thus we hypothesize that the effect (if it exists) of this polymorphism might be contributing with other factors to give rise to aneuploidy. It has been proposed that non-disjunction involves several “hits” at different stages of development [41]. The non-disjunction of homologous chromosomes in meiosis I is linked with the absence of recombination and it is not necessarily associated with advanced maternal age [41,43]. It has been proposed that there are factors that affect the accessibility of chromosomal regions to recombine, which results in abnormalities in chromosome segregation [44]. It is possible that alterations in sequences that are important for the three-dimensional (3D) positioning of acrocentric chromosomes, such as D15Z1 polymorphism, could represent one of these “hits” that alter the accessibility of chromatin for recombination, resulting in altered disjunction of acrocentric chromosomes. Interestingly, this possible mechanism would not imply the co-segregation of the chromosome carrying the polymorphism and the non-disjointed chromosome, which would explain the lack of an increased frequency of the polymorphism in the aneuploid abortions and offspring. Alternatively, it is possible that the presence of the D15Z1 polymorphism and non-disjunction are related to a common trigger, such as exposure to environmental pollutants [45,46,47]. We recognize that our cohort is small in size; therefore, further studies are required to verify the influence of this polymorphism in non-disjunction.

The intriguing D15Z1 variant could also be giving us a lesson on present-day evolution of human chromosomes. The short arms of the human acrocentric chromosomes, where D15Z1 is located, consist of short highly repetitive heterochromatic sequences that form higher-order-repeats units arranged in tandem. This heterochromatic region surrounds clusters of the 45S rDNA, known as NOR, which are located in the p12 region of acrocentric chromosomes. In human, 10 NOR exists that are located on the 10 acrocentric chromosomes of a diploid cell. The NOR form the secondary constriction in metaphase chromosomes and the nucleoli in the interphase nucleus. During interphase, the heterochromatin organizes NOR regions in large clusters with high transcriptionally active rDNA surrounded by heterochromatin in a perinucleolar position, thus rendering only one to three observable nucleoli.

Satellite DNA plays an important role in the organization of the nucleolar architecture and its transcriptional activity, as demonstrated by the erroneous position of the nucleolus when rDNA arrays do not contain the proximal and distal beta satellite DNA sequences, known as DJ and PJ (Figure 1) [18,25]. The importance of the integral function of rDNA and its perinucleolar heterochromatic sequences is probably the reason why the immediately proximal and the distal sequences to rDNA have been conserved across all acrocentric chromosomes [18,26]. The most heterogeneous family is that proximal to the centromere, being formed by satellite III DNA. This region contains highly repetitive units that are made of two sequence motifs, GGAAT and GGAGT, and, depending on the percentage of these two motifs and the diversification of their sequence, two groups are recognized: group 1 comprising the subfamilies pTRS-63, pW-1, pK-1 and PE-1; and group 2 with pTRS47, pE-2, pR-1, pR-2 and pR-4. All of these subfamilies are shared by more than one acrocentric chromosome (Figure 1), suggesting that the chromosome’s acrocentric short arms have evolved in a concerted manner.

As a consequence of interchromosomal exchanges between the rDNA clusters, it might be expected that sequences distal to rDNA would be shared by all acrocentrics and the more proximal sequences may display more chromosome-specific sequences [17]. However, the only chromosome-specific sequence is D15Z1 (which belongs to group 1), being located at distal 15p; whereas, other sequence subfamilies are shared by all acrocentric chromosomes and they are highly homogeneous (Figure 1). The high degree of homogenization of sequences of acrocentric chromosomes p arms, as compared to non-acrocentric chromosomes, is usually caused by recombination between non-homologous chromosomes, and it is an important mechanism for concerted evolution [19].

The high frequency of the D15Z1+ polymorphism in acrocentric chromosomes other than 15 could suggest that we are witnessing a phenomenon of sequence homogenization across acrocentric chromosomes. It is not clear whether mobilization of D15Z1 is carried out through unequal crossing-over between non-homologous chromosomes, by gene conversion or by translocation between acrocentric short arms, since all of these mechanisms are favored if some degree of sequence homology exists between the donor and receptor sequences. Practically all subfamilies of satellite III DNA share different degrees of homology [19,26]; however, given the frequency with which chromosomes 13 or 14 are the acceptor chromosomes for sequence D15Z1, we would expect a higher degree of sequence homology among the chromosomes belonging to group D. 

According to Bandyopadhyay [19], in the absence of a strong driving force, the satellite III DNA sequences may have a very slow process of interchromosomal transfer. However, if physical permissibility exists, the transference might result in directional spreading of a variant. The complex organization of the nucleolus during interphase could spatially locate chromosomes, 13, 14, and 15 in an interaction that is permissive for the mobilization of the D15Z1 region, which is practically the only known chromosome-specific region, which has not initiated a homogenization. The high frequency of homozygous individuals for the 13pvar variant (D15Z1+) found in our population and the high frequency of homozygous individuals for the 14pvar variant (D15Z1+) found by Cockwell [24] could reflect a concerted evolution step related to the important function of the centromere and the NORs, which flank satellite III DNA. In any case, the observation of this chromosomal variant across the acrocentric chromosomes might indicate an ongoing process of sequences homogenization of the short arms of this group of chromosomes.

The high mobilization rate of the acrocentric satellite III, and especially of the D15Z1 region, is inferred by the high frequency with which this sequence is involved in the breakpoints of chromosomal rearrangements, (such as Robertsonian translocations and supernumerary marker chromosomes) [48], and its involvement in the generation of chromosomal structural rearrangements [2,49]. Thus, this chromosomal variant can generate chromosomal alterations of the structural type and, according to our data, a possibility exists that D15Z1 might also boost the frequency of numerical alterations. Interestingly, all of these phenomena could be an after-effect to the transfer of sequences between acrocentric chromosomes to preserve the structure and function of the nucleolus, part of the ongoing concerted evolution of the human chromosomes.

## 4. Conclusions

Our results indicate that the D15Z1 region is located on a chromosome other than 15 at an overall frequency of 21.6%, which shows that this polymorphism is present in ethnicities as diverse as Caucasian and Mexican mestizo at similar frequencies.

We found an increased frequency of polymorphism in individuals with aneuploid offspring; however, more research is required to establish whether there is a causal relationship or whether it is an association related to a third common factor.

The high frequency of the polymorphism could suggest that the high mobility of the region is contributing to a sequence homogenization of the acrocentric p arms, related to the important function of the centromere and the nucleolar organization region, which flank satellite III DNA.

## 5. Materials and Methods

### 5.1. Patient Cohorts

We included 75 couples with healthy offspring and 10 of their children, 87 couples who reported a child or miscarriage with aneuploidy for any of the acrocentric chromosomes, and 73 of their children with regular trisomy 21, for a total of 407 individuals. The research and ethics committees of the National Institute of Pediatrics, México, with the project code 2009/89, approved this project. All couples signed an informed consent letter, in which they agreed to voluntarily participate in the study and allowed or refused the inclusion of their children in the project. All of the participants were studied by GTG banding karyotype and by FISH for detecting the number and localization of the D15Z1 region in all of the chromosomes; in addition, DAPI fluorescent bands were used to identify all of the involved chromosomes according to the q arm banding pattern.

### 5.2. Cytogenetic Study with GTG bands

Peripheral blood samples were obtained from parents with healthy children, from parents with aneuploid children and from healthy or aneuploid children. Whole blood was cultured in RPMI medium (Invitrogen, California, USA), supplemented with 1% of phytohemagglutinin (Invitrogen, USA), and incubated for 72 h at 37 °C. Colchicine (0.08 g/mL) (Sigma, San Luis Missouri, USA) was added for the last two hours of culture to obtain metaphase spreads. Harvesting was made using hypotonic solution of 0.075M KCl (Sigma, USA), and cold Carnoy solution (Methanol: Acetic acid, 3:1), then cells were dropped on cold slides and immediately placed on a thermal plate at 60 °C; GTG banding was performed and 20 metaphases per individual were analyzed, at a resolution of 450–550 bands per haploid set. In cases where the gain or loss of a chromosome was observed in one cell, the analysis was extended to 50–100 cells to rule out or confirm mosaicism, according to the criteria of the International Cytogenetic Nomenclature System [50]. Slides were blind coded by a person not involved in the study.

### 5.3. Fluorescence In Situ Hybridization

For all participants, a karyotype was obtained by analyzing twenty metaphases with GTG banding per individual in a light field microscope. Fluorescence in situ hybridization (FISH) was performed with a cocktail containing the probe D15Z1 (Vysis, IL, USA) in aqua, as well as the centromeric probes 13/21 (D13Z1/D21Z1) in green (Kreatech, Amsterdam, NL), and 14/22 (D14Z1/D22Z1) in red (Kreatech, Amsterdam, NL, USA) to detect the presence and localization of the D15Z1 region. Cells were dripped in steam, which ensured that the metaphases had the least amount of cytoplasm. The slides were treated with pepsin (0.1 mg/mL), then fixed with formaldehyde, dehydrated, and codenatured at 73 °C for 2 min.; hybridized at 37 °C for 24 h and contrasted with DAPI/Vectashield (Vector, Peterborough, UK). Post-hibridization washes were performed at high stringency while using a solution containing 0.4XSSC/0.3% NP40 at 70ºC ± 1ºC for two minutes and in 2XSSC/0.1% NP40 at room temperature for one minute. Metaphase spreads were analyzed using the Imager-Zeiss microscope with ISIS software.

### 5.4. FISH Analyses

Thirty metaphases per individual were analyzed. Over 90% of the metaphases had the complete set of acrocentrics; however, we also included spreads with at least 45 chromosomes; all chromosomes were identified through the DAPI banding pattern. For analyses of the fluorescent probes, images were acquired with three single filters, green, red, and aqua, and the three images were merged into a single image. Acrocentric chromosomes were identified by analyzing the DAPI banding pattern and by the fluorescent centromeric probes. These analyses permitted the unequivocal identification of every chromosome pair and the localization of the D15Z1 within the short arms of all acrocentric chromosomes.

### 5.5. Statistical Analyses

A Fisher´s Exact Test of two tails performed the comparison of the extra D15Z1 signal frequency between the groups and the analysis of the Hardy–Weinberg equilibrium was completed by the Fisher´s Exact Test using de Finneti software. The association of the extra D15Z1 signal between parents of healthy children and parents of aneuploid offspring was tested by the Armitage´s trend test with the de Finneti software [51,52]. A *p* < 0.05 was considered statistically significant.

## Figures and Tables

**Table 1:** Abnormal karyotypes in four individuals from the two groups of couples.

Group	GTG ^1^ Karyotype	Carrier of D15Z1 Polymorphism
Couples with healthy offspring	mos 45,X[4]/46,XX[89]	-
	46,XX,inv(8)(q13q24.1)	-
Couples with aneuploid offspring	mos 47,XY,+21[3]/46,XY[62]	-
	mos 45,X[3]/46,XY[40]	13pvar(D15Z1+)

^1^ Giemsa-Trypsin G banding.

**Table 2:** Presence of extra D15Z1 signals according with the Mother age.

Group	*n*	Age ± SD	Mothers Aged ≤34	Mothers Aged ≤34 with Extra D15Z1	Mothers Aged ≥35	Mothers Aged ≥35 with Extra D15Z1
Mothers with healthy offspring	75	25.9 ± 5.3	72	14 (19.4%) ^1^	3	0 (0%) ^2^
Mothers with aneuploid offspring	87	30.4 ± 7.3	61	15 (24.6%) ^1^	26	5 (19.2%) ^2^

^1^*p* = 0.68 Fisher’s Exact Test (two tails) ^2^ *p* = 1.0 Fisher’s Exact Test (two tails).

**Table 3:** Distribution of D15Z1 polymorphism in individuals from couples with healthy or aneuploid offspring.

Individuals	*n*	With Extra D15Z1	%	More than One Extra D15Z1 ^1^
With healthy offspring	150	21/150 ^2^	14	4/21
With aneuploid offspring	174	46/174 ^2^	26.4	4/47
Total	324	67/324	20.7	8/68

^1^ Four individuals of each group presented more than one extra-signal D15Z1, either in both chromosomes 13p or one signal on 13p and other on 14p or 21p. ^2^ *p* = 0.022 Fisher’s Exact Test (two tails).

**Table 4:** Individuals with an extra D15Z1 region and distribution of the additional D15Z1 signal among the acrocentric chromosomes by group of patients.

Individuals	Carriers Extra D15Z1 (%)	D15Z1 Extra-Signal Receptor Chromosomes (%)									
		Single Extra Signal				Multiple Extra Signals					
		13	14	21	22	13 × 2	13 × 2 +14	13 + 14	13 + 21	14 × 2	14 + 21
With healthy offspring *n* = 150	21 (14)	6/21 (28.6)	10/21 (47.6)	0/21	1/21 (4.8)	3/21 (14.3)	0/21	0/21	1/21 (4.8)	0/21	0/21
Healthy offspring *n* = 10	5 (50%)	1/5 (20)	3/5 (60)	0/5	0/5	0/5	0/5	1/5 (20)	0/5	0/5	0/5
With aneuploid offspring *n* = 174	46 (26.4)	28/46 (60.8)	10/46 (21.7)	4/46 (8.7)	0/46	0/46	1/46 (2.2)	1/46 (2.2)	0/46	1/46 (2.2)	1/46 (2.2)
Aneuploid offspring *n* = 73	16 ^1^ (21.9)	8/16 (50)	3/16 (18.75)	2/16 (12.5)	0/16	1/16 (6.25)	0/16	2/16 (12.5)	0/16	0/16	0/16
Total *n* = 407 Frequency per individual	88/407 (21.6) ~1/5	43 (10.6) 1/10	26 (6.4) 1/16	6 (1.5) 1/68	1 (0.25) 1/407	4 (1.0) 1/102	1 (0.25) 1/407	4 (1.0) 1/102	1 (0.25) 1/407	1 (0.25) 1/407	1 (0.25) 1/407

^1^ Among the 16 children studied, 15 came from 33 couples (out of a total of 39) who were carriers of polymorphism and one came from a non-carrier couple and was considered a probable *de novo* event.

## Abbreviations

DA/DAPI	Distamycin A/4,6-diamino-2-phenyl-indole
DAPI	4’,6-diamidino-2-phenylindole
FISH	Fluorescence In Situ Hybridization
GTG	Giemsa-Trypsin G banding
ISCN	International System for Human Cytogenomic Nomenclature
NOR	Nucleolar organization region
rDNA	Ribosomal DNA, Clusters of DNA sequences containing repeated units of rRNA genes
rRNA	Ribosomal ribonucleic acid
